# A Case of Abscess of the Liver, with a Formation of Hydatids

**Published:** 1803-12-01

**Authors:** 

**Affiliations:** Derby


### *A Case of* Abscess of the Liver, *with a Formation of* Hydatids; *communicated by* Doctor Robinson, *of Derby.*

On March 30th, I was desired to see Mrs. T. at Stoke upon Trent, who appeared much emaciated, and laboured under a fully formed hectic fever, attended with an almost constant nausea and frequent vomiting, especially when she attempted to take any thing solid. In the region of the right hypochondrium there was a very considerable tumour, extremely painful to the touch, and giving, upon pressure, an evident sense of fluctuation. Mr. Beaver, the attending surgeon, and an ingenious practitioner at Stoke, observed that this enlargement began about ten days before, when he ordered leeches to be applied to the part with a perpetual blister, and two grains of calomel, five grains of hemlock, and six of rhubarb, to be taken every night.

Mrs. T. is about thirty years of age, of a delicate habit, and has led a very temperate life, but from her sixteenth year has been subject to repeated attacks of jaundice; has always been costive, and sometimes has passed ten days without having had any evacuation from the bowels. Several of her family have suffered under affections of the liver, and Mrs. T. has discovered a hardness and fulness in the region of the right hypochondrium for several years. For about two months before I saw her, she had frequently experienced an acute pain in this part, which was generally relieved by camphor, opium, and æther, ordered by Mr. Beaver; blisters had also been applied, and pills consisting of calomel, aloes, and Castile soap, taken as occasion required.

The evident marks of a formation of matter being present when I was sent for, together with the conical shape and pointed appearance of the tumour, made me hope that the abscess would burst externally; and to encourage this this, I directed emollient cataplasms to be applied, and fomentations to be used; at the same time, a pill consisting of calomel and digitalis, of each half a grain, was ordered to be taken every night and morning; and a draught, consisting of tinct. of columbo, antimonial wine, and Minderus's spirit, was prescribed three times a day. For common beverage the patient drank the nitrous acid and water. Perceiving, after a few days, that the tumour, in place of coming more to a head, receded, and appeared to extend more towards the cavity of the abdomen, I thought it most prudent to have it opened, which operation Mr. Beaver performed by making an incision, one inch in length, in the most depending part of the tumour. Rather more than two pints of purulent matter were discharged, with immediate relief. The exhibition of the submuriate of quicksilver, &c. was now suspended, and she was directed a light, nutritious diet; and took every night at bed time, extract of henbane and antimonial powder, of each two grains. For about a week or ten days she regained strength, and appeared to be doing as well as possible, when the matter suddenly ceased to flow from the abscess, and she again experienced extreme acute pain, with sense of fulness, in the part; yet Mr. B. upon examination with a probe, could not perceive any obstruction in the opening. About three days after this, a large hydatid made its appearance, followed by several others, varying in size from a pigeon's egg to a small pea; a considerable quantity of purulent matter was also again discharged.

At this time I was again sent for; but from the violence of the hectic fever, together with the increasing debility and irritability of the system, I entertained but slight hopes of her recovery; I ordered, however, a solution of half a grain of the muriate of quicksilver in about four ounces of warm milk, with a small portion of tinct. of myrrh, to be injected into the abscess once a day, and one drachm of the strong mercurial ointment to be rubbed in the right hypochondrium every night. The hydatids, after a few days, gradually disappeared, and the matter discharged became much tinged with bile, but which afterwards, by degrees, assumed the appearance of healthy pus. Nothing further was now done, except keeping the abscess open by means of a sponge tent; and our patient, with but slight interruptions, continued to regain her health and strength, which I believe she still continues to enjoy, with more comfort than she has done for many years past.

*Derby, Nov.* 3, 1003.

